# Fe, N-Doped Metal Organic Framework Prepared by the Calcination of Iron Chelated Polyimines as the Cathode-Catalyst of Proton Exchange Membrane Fuel Cells

**DOI:** 10.3390/polym13213850

**Published:** 2021-11-08

**Authors:** Yu-Wei Cheng, Wen-Yao Huang, Ko-Shan Ho, Tar-Hwa Hsieh, Li-Cheng Jheng, Yang-Ming Kuo

**Affiliations:** 1 Department of Chemical Engineering, Ming Chi University of Technology, New Taipei City 24301, Taiwan; louischengblue@gmail.com; 2 Department of Photonics, National Sun Yat-Sen University, 70 Lienhai Rd., Kaohsiung 80424, Taiwan; wyhuang@faculty.nsysu.edu.tw; 3 Department of Chemical and Materials Engineering, National Kaohsiung University of Science and Technology, 415, Chien-Kuo Road, Kaohsiung 80782, Taiwan; lcjheng@nkust.edu.tw (L.-C.J.); kuoyang1228@gmail.com (Y.-M.K.)

**Keywords:** FeNC catalyst, polyimine, two-stage calcination, oxygen reduction reaction

## Abstract

Aromatic polyimine (PIM) was prepared through condensation polymerization between p-phenylene diamine and terephthalaldehyde via Schiff reactions. PIM can be physically crosslinked with ferrous ions into gel. The gel-composites, calcined at two consecutive stages, with temperatures ranging from 600 to 1000 °C, became Fe- and N-doped carbonaceous organic frameworks (FeNC), which demonstrated both graphene- and carbon nanotube-like morphologies and behaved as an electron-conducting medium. After the two-stage calcination, one at 1000 °C in N_2_ and the other at 900 °C in a mixture of N_2_ and NH_3_, an FeNC composite (FeNC-1000A900) was obtained, which demonstrated a significant O_2_ reduction peak in its current–voltage curve in the O_2_ atmosphere, and thus, qualified as a catalyst for the oxygen reduction reaction. It also produced a higher reduction current than that of commercial Pt/C in a linear scanning voltage test, and the calculated e-transferred number reached 3.85. The max. power density reached 400 mW·cm^−2^ for the single cell using FeNC-1000A900 as the cathode catalyst, which was superior to other FeNC catalysts that were calcined at lower temperatures. The FeNC demonstrated only 10% loss of the reduction current at 1600 rpm after 1000 redox cycles, as compared to be 25% loss for the commercial Pt/C catalyst in the durability test.

## 1. Introduction

The oxygen reduction reaction (ORR) is usually the bottleneck reaction for fuel cells, implying that catalysts, which are usually precious and expensive, are needed to lower the barrier of the reaction in order to improve the power and productivity of the fuel cells. To carry out ORR in a cheaper way without depressing the catalyzing capability of Fe, N-doped MOF (metal organic framework) composites are prepared, in which covalent-bonded iron nitrogen (Fe–N) can become an active center in the carbonaceous matrix after calcination.

The first MOF-based cathode catalyst was prepared using cobalt-coordinated with large cycled phthalocyanine [1], which was, over the following year, modified via high-temperature calcination to become Co-porphyrin. This did not increase the efficiency of the catalyst; however, the ORR in the cathode improved significantly [2]. Eventually, it was understood that calcination at a higher temperature than 800 °C is required to obtain an MOF-based cathode catalyst. Some iron- and nitrogen -containing carbonaceous materials [3] were calcined in the presence of N_2_ or NH_3_ to create the micro- or mesoporous areas of FeNC with defined numbers of active sites. FeNC was made available after calcination at temperatures that were higher than 950 in the argon and ammonia atmosphere in a study conducted by Mamtani [4]. Several closely related works for ORR and MOF can be found. Refs. [5,6,7,8] In a conventional iron- and nitrogen-doped MOF (FeNMOF), Fe ions are designed to be captured (complexed) with multi-nitrogen (usually 4-nitrogen) in a cyclic compound that contains 4-nitrogens [9,10,11,12,13] before being subjected to calcination. However, the preparation of FeNMOFs requires many tedious series of steps of organic reactions with limited yield. In other words, the expense of obtaining FeNMOF is close to that needed to purchase precious metals such as Pt or Pd. In this study, we attempted to avoid the tedious steps of organic synthesis and the associated expenses by directly polymerizing a nitrogen-containing polymer (polyaniline: PANI) in the presence of either Fe or Co ions to allow ions to complex with the nitrogen-containing monomers before the initiation of polymerization. New publications [14,15] on the application of PANI in the design of biosensors and biofuel cells are available. The polymerization of PANI on the carbonaceous surfaces demonstrated significant ORR in both acidic and alkaline media [16,17,18]. Additionally, the amino groups of the N-containing PANI on an XC72 (Vulcan) support could coordinate with Pt ions, resulting in the presence of a well-dispersed Pt-catalyst on the XC72 surfaces and the control of the pore sizes of the obtained PANI/XC72 composites [19,20]. However, PANI can easily be prepared by polymerizing aniline monomers (NH_4_^+^) in the acidic aqueous solution [21,22,23,24]. The repulsive force between NH_4_^+^ and Fe^+2^ or Co^+2^ hinders the coordination (complex) and decreases the amounts of metal ion captured by the resultant PANIs. Furthermore, due to the steric effect of the two huge benzene rings located on both sides of the amino group (-NH) in the backbone of PANI, most of the metal ions are not able to come close enough to induce the complexation with -NH, even when the PANI was synthesized following polymerization. Therefore, the degree of coordination with the metal ions by PANIs was too low to become an efficient cathode catalyst of fuel cell after calcination. Consequently, additional small N-containing molecules such as ethylene diamine (EDA) were added to capture most of the metal ions firmly in water before the addition of anilinium monomers to increase the degree of coordination before and after polymerization [25].

In this study, aromatic polyimine (PIM) replaced PANI to effectively remove the steric hindrance and allow the approach of metal ions to form robust coordination bonding before calcination, without the necessity of adding any small N-containing molecules before polymerization and calcination. Since PIM can usually be prepared quickly with a high yield, at temperatures slightly higher than room temperature, by means of Schiff condensation between the diamine and the dialdehyde, we used p-phenylene diamine (PDA) and terephthaldehyde (TPAl) as the monomers to obtain aromatic PIM. Compared to PANI, it was found that there was a large empty space around the imine groups (-N=CH-) without the presence of a huge benzene ring at one end, and that no hydrogen was connected to the nitrogen of the imine. Furthermore, no positive charge was present on PDA and TPAl monomers to repulse the positive metal ions before or after polymerization. Theoretically, the addition of metal ions could be carried out before polymerization with monomers or after polymerization with polymers, both of which could create a physically crosslinked gel if the degree of complexation is high enough. In other words, we were able to judge the degree of complexation according to whether or not gel was formed prior to being subjected to calcination.

## 2. Materials and Methods

### 2.1. Materials

p-Phenylene diamine (PDA) (Tokyo Kasei Kogyo Co., Ltd., Tokyo, Japan), terephthaldehyde (TPAl) (Tokyo Kasei Kogyo Co., Ltd., Tokyo, Japan), and iron(II) chloride hexahydrate (FeCl_2_·6H_2_O, J.T. Baker, NJ, USA) were used in this study.

### 2.2. Preparation of FeNC Catalyst

Quantities of 1.34 g of PDA and 1.62 g of TPAl were placed in 80 and 50 mL of alcohol, respectively, before being mixed into a single solution. The mixture solution was stirred at room temperature for 12 h, during which the color changed to thick orange, indicating that the polymerization is complete. Then, 0.04 g of Iron(II) chloride hexahydrate was introduced into the solution and the viscosity of the mixture gradually increased before turning into a frozen gel. The gel-like composite was concentrated by centrifugation at 300 rpm for 10 min to obtain the precipitate in the bottom of the centrifugation tube. The precipitate was dried at 80 °C for 8 h before cooling to RT.

**Scheme 1:**
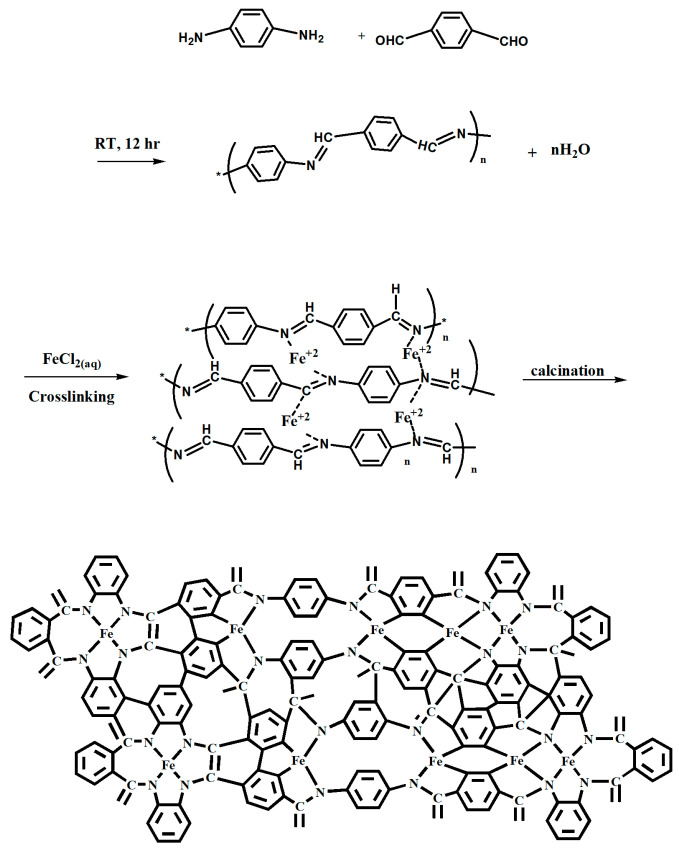
Preparation diagram of PIM and FeNC catalyst.

The obtained PIM, which was the precursor of the FeNC catalyst, was heated to 600 °C (700, 800, 900, 1000 °C) at 10 °C min^−1^ and maintained at 600 °C (700, 800, 900, 1000 °C) for 1 h in the argon atmosphere, then cooled to room temperature. The impurities and magnetic parts of the obtained materials were removed via washing in 9 M H_2_SO_4_ (aq.) at 80 °C for 36 h, followed by filtration, and the cake was washed with de-ionized water and alcohol before drying in a vacuum oven at 80 °C for 8 h. The acid-leached products were further calcined at 500 °C (600, 700, 800, 900 °C) in N_2_ and NH_3_ atmospheres, at 10 °C min^−1^ (named as FeNC-600A500), and washed again in 1 M H_2_SO_4_ (aq.) at 80 °C for 3 h, followed by drying in a vacuum oven at 60 °C. The sample was named FeNC-600A500 (-700A600, -800A700, -900A800, and -1000A900). The schematic diagram depicting the preparation of the FeNCs is shown in Scheme 1.

### 2.3. FTIR Spectroscopy

The main functional groups of PDA, TPAl, and PIM were assigned in accordance with the FTIR spectra that were recorded on an IFS3000 v/s FTIR spectrometer (Bruker, Ettlingen, Germany) at room temperature with a resolution of 4 cm^−1^ and 16 scanning steps.

### 2.4. X-ray Photoelectron Spectroscopy (XPS)

The different binding energy spectra of N1s of various FeNCs were used to characterize the percentage of nitrogen in pyridine, pyrrole, graphenec, Fe-N., etc. after calcination with an XPS instrument produced by Fison (VG)-Escalab 210 (Fison, Glasgow, UK) using Al Ka X-ray source at 1486.6 eV. The pressure in the chamber was kept at 10^−6^ Pa or less during the measurement. The powered samples were shaped to become tablet samples using a stapler. The binding energies of the N1s around 400 eV were recorded.

### 2.5. Wide Angle X-ray Diffraction: Powder X-ray Diffraction (WXRD)

A copper target (Cu-Kα) Rigaku x-ray source (Rigaku, Tokyo, Japan), with a wavelength of 1.5402 Å, was the target for x-ray diffraction. The scanning angle (2θ) ranged from 10 to 90°, with a voltage of 40 kV and a current of 30 mA, and was operated at 1° min^−1^.

### 2.6. Scanning Electronic Microscopy (SEM)

Using a SEM (field emission gun scanning electron microscope, AURIGAFE, Zeiss, Oberkochen, Germany), the sizes and morphologies of the FeNCs were obtained.

### 2.7. Transmission Electronic Microscopy (TEM)

Photos of the samples were taken using an HR-AEM field-emission transmission electron microscope (HITACHI FE-2000, Hitachi, Tokyo, Japan); the samples were dispersed in acetone, and were subsequently placed dropwise on carbonic-coated copper grids before being subjected to emissions.

### 2.8. Surface Area and Pore Size Measurement (BET Method)

Nitrogen adsorption–desorption isotherms (type IV) were obtained from an Autosorb IQ gas sorption analyzer (Micromeritics-ASAP2020, Norcross, GA, USA) at 25 °C. The samples were dried in a vacuum overnight at a temperature above 100 °C. The surface area was calculated according to the BET equation when a linear BET plot with a positive C value was in the relative pressure range. The pore size distribution was determined according to methods derived from the Quenched Solid Density Functional Theory (QSDFT), based on a model of slit/cylinder pores. The total pore volumes were determined at P/P_0_ = 0.95.

### 2.9. Electrochemical Characterization

#### 2.9.1. Current–Potential Polarization-Linear Scan Voltammetry (LSV)

The performance of the electrocatalyst support was implemented in a three-electrode system. The round working electrode, which had an area of 1.5 cm^2^, was prepared as follows: Ag/AgCl, carbon graphite, and a Pt-strip were used as the reference, relative, and counter electrode, respectively. The electrochemical test was carried out in a potentiostat/galvanostat (Autolab-PGSTAT 30 Eco Chemie, KM Utrecht, The Netherlands) in 0.1 M HClO_4_ solution, and C-V curves were obtained with scanning potentials from −0.2 to 1.0 V at a scanning rate of 50 mV·s^−1^. The catalyst ink was prepared by mixing 3 mg support powder in isopropanol and stirring until it became uniform. Subsequently, 5% Nafion solution was added into the mixture as a binder, the mixture was ultra-sonicated for 1 h, and the obtained ink was uniformly spray-coated on the carbon paper for C-V testing.

The current-potential polarization curves obtained from LSV of the various FeNCs were measured using a rotating-disk electrode (RDE: Metrohm, FL, USA) operating at 900, 1200, 1600, 2500, and 3600 rpm in O_2_-saturated 0.1 M HClO_4_, respectively. The reduction current densities of various FeNCs, which were recorded at 1600 rpm within the measured voltage range (0.0~1.2 V), were chosen for comparison.

#### 2.9.2. MEA Preparation

A Nafion^®^ 212 sheet, purchased from Ion Power Inc., New Castle, DE, USA, was used as the proton exchange membrane. To remove the surface organic impurities and to convert the membranes into protonated (H^+^) form, the Nafion-212 (4 × 4 cm), membrane was treated at 70 °C in 5 wt.% H_2_O_2_ aqueous solution for 1 h, and was then submerged in 1 M H_2_SO_4_ solution for 1 h. Subsequently, the treated membranes were dipped in distilled water for 15 min and were then stored in deionized water. The catalyst inks were prepared by mixing 20 mg of FeNC powders in isopropanol and were mechanically stirred until they became uniform, followed by the addition of 5% Nafion solution, before stirring again to reach uniformity. Eventually, the catalyst mixture was ultra-sonicated for 1h, followed by dropwise coating on both sides of the treated Nafion sheet, as the anode and cathode electrodes (2 × 2 cm), respectively, and hot-pressing at 140 °C with a pressure force of 70 kg cm^−2^ for 5 min to obtain the MEA.

#### 2.9.3. Single-Cell Performance Testing

The MEA was installed in a fuel cell test station to measure the current and power densities of the assembled single cell using a single-cell testing device (model FCED-P50; Asia Pacific Fuel Cell Technologies, Ltd., Miaoli, Taiwan). The active cell area was 2 × 2 cm^2^. The temperatures of the anode, cell, cathode and humidifying gas were maintained at around 70 °C. The flow rates of the anode input H_2_ and the cathode input O_2_ fuels were set at 200 and 100 mL·min^−1^, respectively, based on stoichiometry. To test the electrochemical performance of FeNC cathode catalyst in the individual MEAs, both the C-V and output powers were measured.

## 3. Results

### 3.1. FTIR Spectra

**Figure 1:**
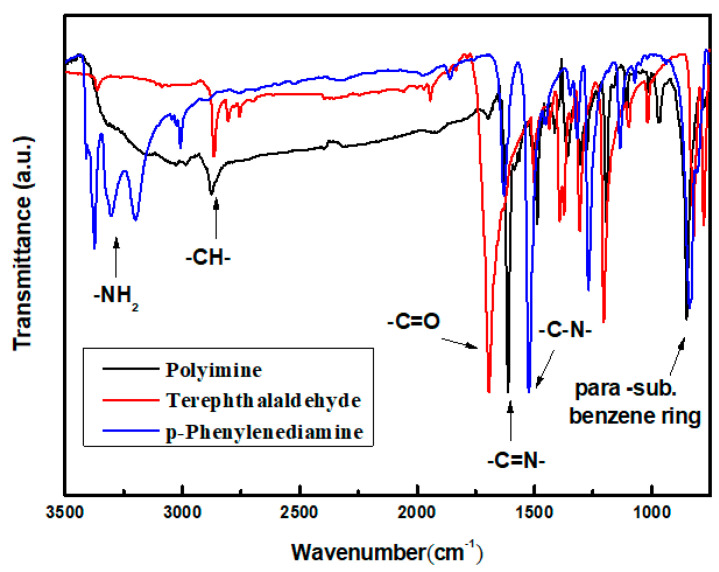
IR spectra of PDA, TPAl, and PIM.

The IR-spectra of the PDA, TPAl monomers, and PIM obtained from the Schiff condensation polymerization are demonstrated in Figure 1. The doublet peaks of the symmetric and asymmetric stretching modes of the primary amine, which belonged to PDA, can be clearly seen at around 3297 and 3201 cm^−1^, respectively. The –C–N– bond is also visible at 1520 cm^−1^ and para-substituted benzene ring contributed to the peak at 835 cm^−1^, which overlapped with the para-substituted ones of TPAl and PIM, indicating that the Schiff reaction was carried out at the para-positions for PDA and TPAl. The carbonyl group of the aldehyde of TPAl contributed the peak at 1700 cm^−1^. The vanishing of the peaks of the carbonyl and primary amine in PIM revealed that the condensation reaction successfully occurred and that water was the by-product. The imine groups of the products of the Schiff reaction caused the sharp peak at 1620 cm^−1^. The related polymerization reaction via Schiff condensation is included in the upper part of Scheme 1.

**Figure 2:**
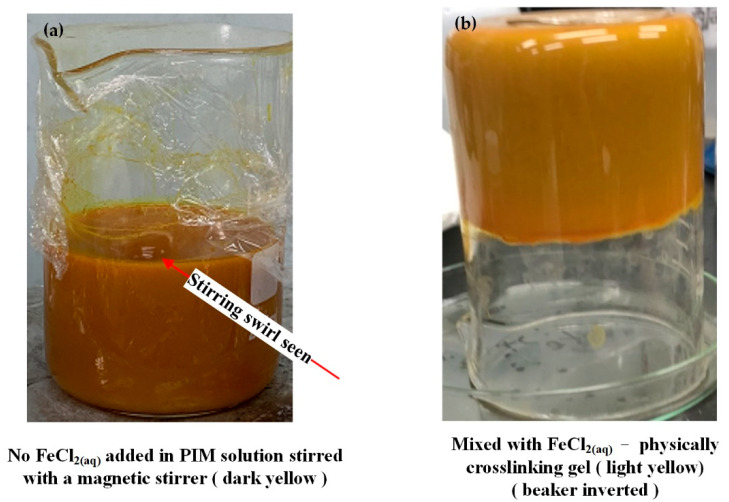
Pictures of PIM alcohol solution (**a**) without FeCl_2(aq)_ and (**b**) with FeCl_2(aq)_.

After heating, the alcohol solution containing a mixture of PIM demonstrated clear swirls during stirring with a magnetic stirrer, as shown in Figure 2a. However, the liquid-like solution gradually started to freeze with the addition of FeCl_2_ and eventually became a gel, as seen in Figure 2b. We concluded that the gel resulted from the formation of physically crosslinked PIM with Fe^+2^ ions, which could easily coordinate with the imine groups belonging to different PIM molecules to build up the crosslinking network of the gel, as depicted in Scheme 1. The gel was eventually calcined in the argon atmosphere to prepare the FeNC (Fe, N-doped MOF), as described in Scheme 1.

### 3.2. XPS

**Scheme 2:**
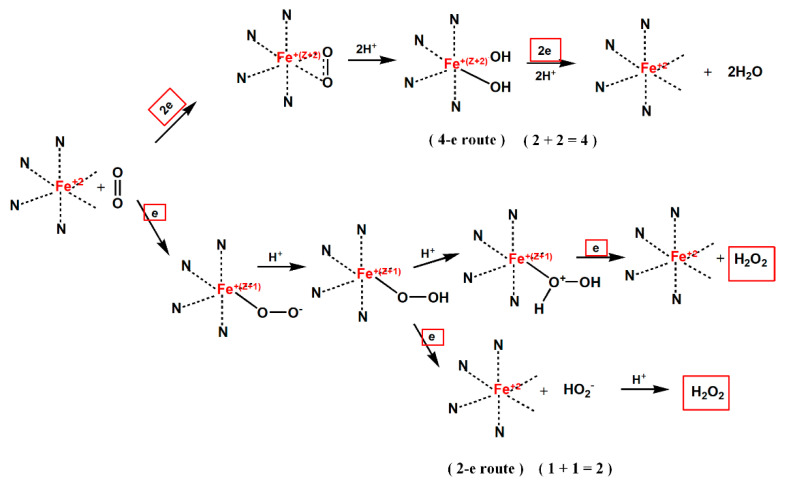
Possible electrocatalytic mechanism by FeNC for ORR.

The active sites of FeNC were able to absorb O_2_ gas and form a peroxide that would dissociate in the presence of protons during reduction (Scheme 2). The formed O_2_-captured Fe-N catalysts were reduced following two approaches [26,27], with one involving the Fe-N catalysts becoming diol and the other involving direct conversion into water following the 4-e route. The captured O_2_ could proceed with another possible ORR with the involvement of two electrons, and H_2_O_2_, not H_2_O, being the final product. The possible formation mechanism of H_2_O_2_, which is illustrated in Scheme 2, reveals that only two electrons were involved. Two possible mechanisms of the formation of H_2_O_2_, depending on the reduction reaction occurring before or after the proton doping, are also described in Scheme 2. The produced H_2_O_2_ could be further reduced to become H_2_O, and an additional two electrons would have become involved if the reduction reaction continued. The catalytic mechanism followed the traditional six-coordinate catalytic reaction for Fe^+2^.

**Scheme 3:**
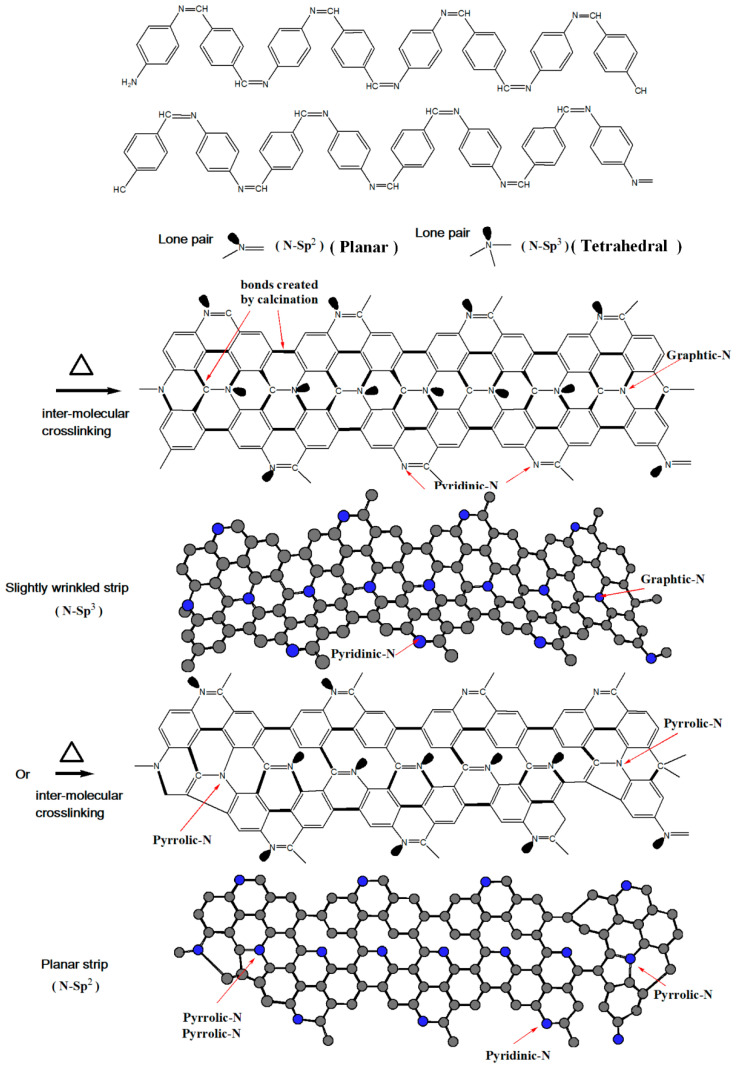
Schematic diagram of the formation of nitrogen-containing active centers in the calcined PIM and N-GF networks.

Theoretically, the O_2_ gas with two lone pairs could be attracted to the active sites of FeNC through the coordination, or could be trapped in the porous holes with various nitrogen-related bonds, in which case the increased polarity of the C-N bonding could improve the O_2_ absorbing capability and cause the C-N bonds to behave as active sites, similarly to transitional metals (Fe). The formation of active nitrogen-containing compounds (–N), such as pyrrolic –N, graphitic –N, and pyridinic –N [28,29,30,31,32,33], is described in Scheme 3. At higher temperatures in the N_2_ atmosphere, the first stage of calcination could create various –N-containing covalent bonds as active sites. In the mixed gases of N_2_ and NH_3_ at lower temperatures, the second calcination could create lots of micro- or mesopores on the FeNC surfaces, resulting in increased surface area and allowing more active –N and –Fe sites for the incoming O_2_ gas.

PIM could crosslink with each other into ladder-like polymers in the initial stage of thermal heating and higher temperature pyrolysis allowed the carbonization between the ladder-like polymers, which could create FeNMOF of graphitic –N, pyridinic –N, and pyrrolic –N (Scheme 3). Most of the pyridinic and pyrrolic –Ns were created on the edges of the calcinated PIM, while graphitic –Ns were mostly formed inside the network. The nitrogen-doped graphene (N-GF)-like structure of the calcined PIM also behaved as a conducting medium, transporting electrons from the anode. This made it possible to avoid the trouble of adding XC-72 during the preparation of the cathode ink. Depending on the sp3 or sp2 bonding of –N- in the aromatic matrix, there were two types of laddered PIMs formed, as illustrated in Scheme 3. For the ladder constructed mainly by sp3 –Ns, the strip of the crosslinked PIM wrinkled slightly, and a more planar strip of the crosslinked PIM formed for sp2 –Ns, as depicted in Scheme 3. The development of the laddered PIM with the increasing of the temperature was able to create an N-GF structure, which is depicted in the bottom of Scheme 3. These GF strips, which were either planar or wrinkled, could self-assemble into thicker slabs, as will be discussed in the SEM section.

**Figure 3:**
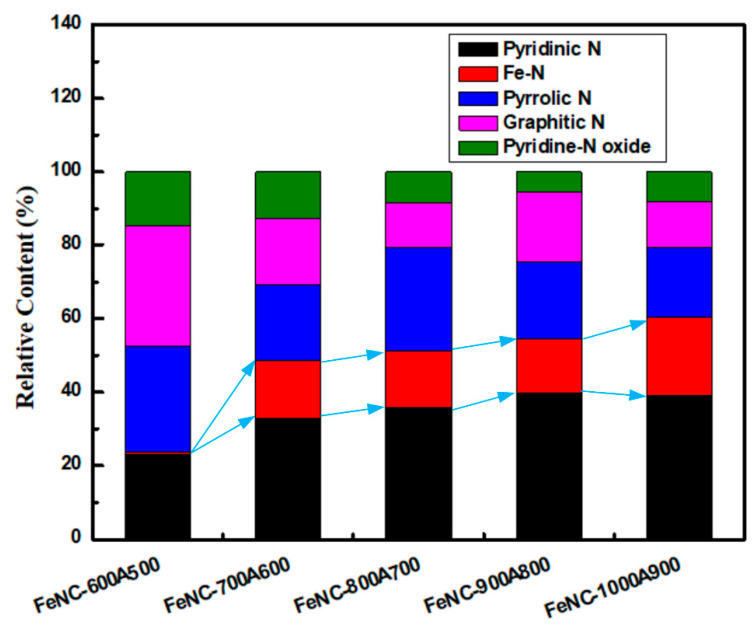
Relative compositions of each type of nitrogen-containing compound found in various FeNC matrices.

The atomic concentration of FeNCs (Fe, N, C, and O) listed in Table 1 clearly demonstrates the increasing of the nitrogen and oxygen atom concentration upon higher temperature calcinations, as measured by XPS. This indicates that more nitrogen could dope into the carbonaceous matrix at higher temperatures, regardless of whether they came from the PIM or the influx of NH_3_ gas, which also caused damage on the catalyst surface and led to an increased surface area, as will be discussed in the BET section. The N_1s_ XPS spectra of FeNCs calcined after acid leaching are presented in Figure S1 and the compositions of each type of nitrogen-doped (-N) group are shown in Figure 3 and Table 2. The covalent-bonded iron and nitrogen (Fe-N) were not found until calcination was higher than 700 °C, and graphitic and pyrrolic –Ns were predominant at temperatures below 600 °C in the second stage of calcination, according to Figure 3, Figure S1, and Table 1. Briefly, more pyridinic –N and active centers of Fe-N (bottom of Scheme 1) bonding were created at the second stage of calcination in the presence of mixed NH_3_ and N_2_ gases. Table 2 also illustrates two major –Ns (pyridinic-N and Fe-N) when the calcination was performed according to the 1000A900 procedure. The increasing temperature created more active sites, which led to a higher LSV current of the cathode and a higher power density of the single cell, which will be discussed in the electrochemical sections.

### 3.3. XRD

**Figure 4:**
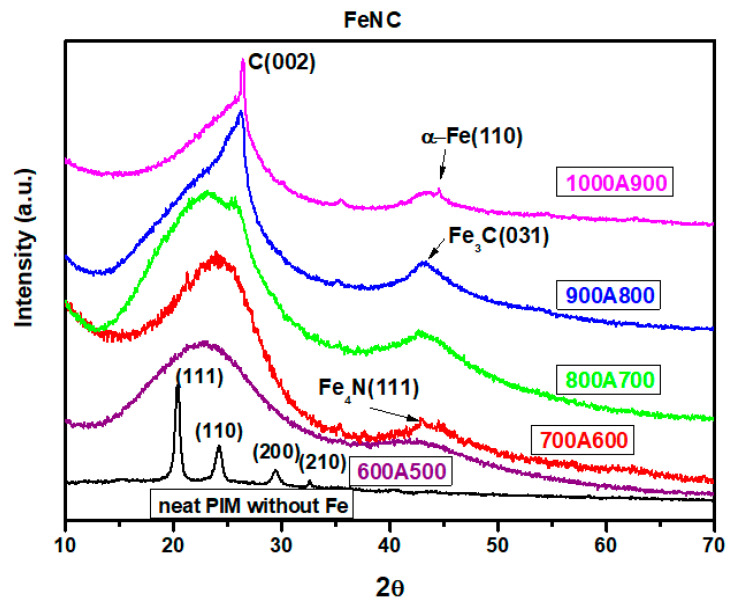
X-ray diffraction patterns of neat PIM and FeNCs prepared with different calcination methods.

The x-ray diffraction patterns, produced through the formation of GF after calcination after 700 °C during the first stage, in which a diffraction peak at 2θ = 26.5° gradually grew with the temperature, are seen in Figure 4. No significant peak is seen at 2θ = 26.5° for neat PIM in Figure 4 except for the characteristic diffraction peaks ((111), (110), (200), and (210)) for pure, aromatic PIM before calcination. The PIM-related crystals were destroyed after 600 °C and only an amorphous pattern remained, demonstrating that the crosslinked PIM (ladder like) did not yet develop into GF or carbon nanotube (CNT) crystals. The Fe was covalently bonded in the amorphous carbon networks at this stage (600 °C), and both the carbonaceous and Fe domains started to create ordered domains after 700 °C, undergoing GF (or CNT)- and Fe-related crystallization (Fe_4_N(111), Fe_3_C(031), α-Fe(110)), respectively. For calcination temperatures over 700 °C, the solid crystallization resulted in the formation of the C(002) plane and more GF (or CNT) crystals started to build up. The characteristic diffraction peak (C(002)) of GF (or CNT) eventually became very sharp at 1000 °C, indicating that the ordered, conducting carbon matrix was entirely formed. Furthermore, the presence of Fe_3_C and α-Fe seeds was able to induce the formation of CNT in the GF-dominating matrix with the increasing of the temperature [34], which will be discussed in the TEM section.

### 3.4. Raman Spectroscopy

**Figure 5:**
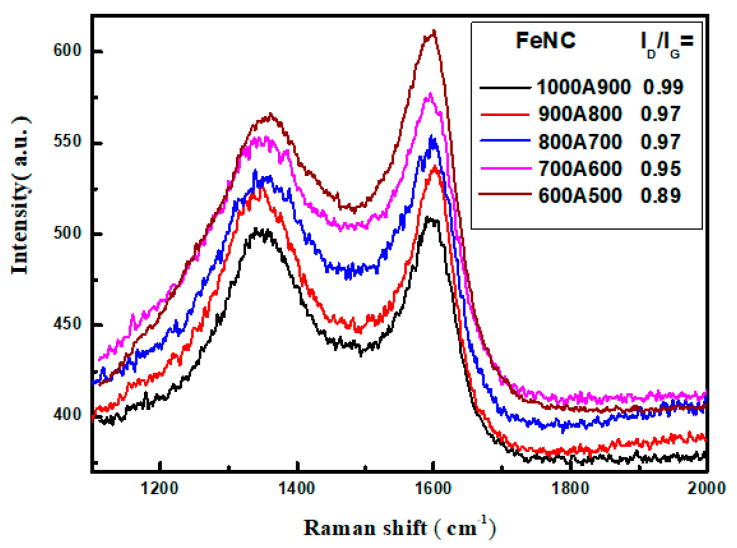
Raman spectra and I_D_/I_G_ ratios of FeNCs prepared with different calcination methods.

Although, as seen in Figure 4, the C(002) plane (2*θ* = 26.5°), which was related to the formation of GF or CNT, became more and more significant with the increasing of the calcination temperature, the intensity of I_G_ (sp^2^) decreased with the temperature, resulting in the increasing of the I_D_/I_G_ ratio in the Raman spectra, as demonstrated in Figure 5. Carbons with sp^2^ bonding outnumbered those with sp^3^ bonding (smaller I_D_/I_G_ ratio) for FeNC-600A500, indicating a more ordered form in their domain, as shown in Figure 5. However, these ordered domains did not contribute to the crystallization, and their x-ray diffraction spectra did not demonstrate significant crystallization peaks, as shown in Figure 4. With the increasing of the calcination temperature for FeNC-700A600, -800A700, the structures of the FeNCs were gradually destroyed by the active, large NH_3_ molecules, which contributed to the increase in I_D_/I_G_ when more sp^2^ bonds were converted to sp^3^ ones after the bombardment of NH_3_ molecules, in accordance with the results shown in Figure 5. It seems that the damage on the structures of FeNCs did not occur on the crystalline region, which developed into GF or CNT at higher calcination temperatures according to the x-ray pattern shown in Figure 4. The robust crystalline structure of the GF (or CNT) formed at high calcination temperatures was able to withstand the attacking of NH_3_ molecules, and to continuously grow into more ordered crystals, as a result of the higher energy provided at higher temperatures. In other words, at high temperatures, the active NH_3_ molecules could only create more surface area for the FeNCs by destroying the amorphous part on the surface (see BET section); the conversion of sp^2^ bonds to sp^3^ but not cause any damage in the crystalline region, which could possibly have been located inside of the matrix.

### 3.5. SEM

**Figure 6:**
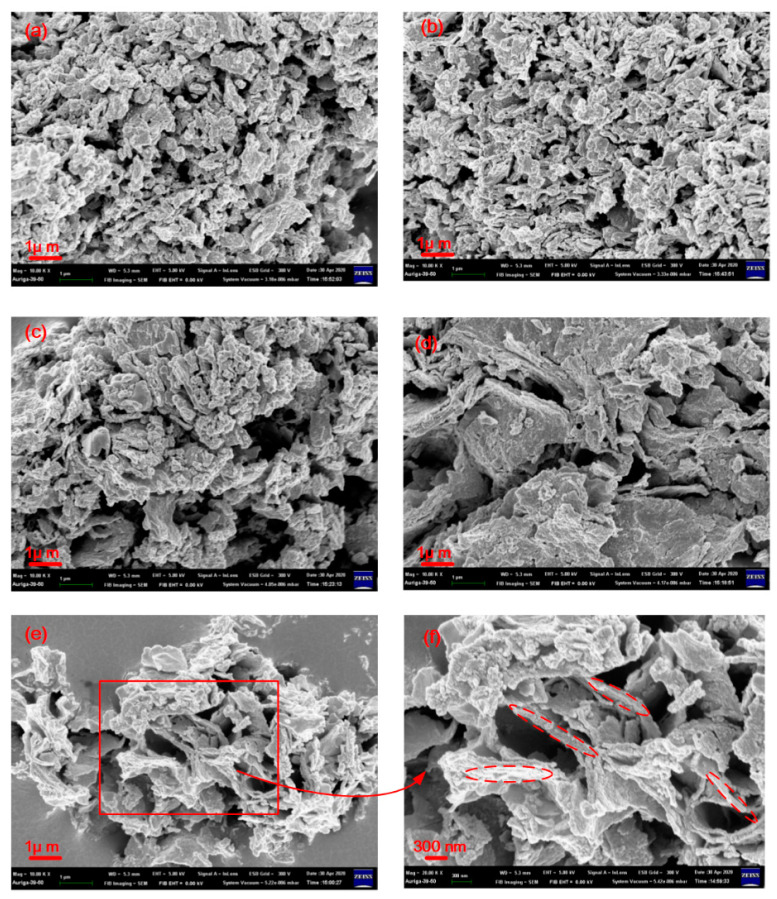
SEM micrographs of FeNCs: (**a**) 600A500, (**b**) 700600, (**c**) 800A700, (**d**) 900A800, (**e**) 1000A900, (**f**) enlarged image of 1000A900.

Only particles with disordered surfaces and short rods are perceivable in the SEM micrographs of FeNC-600A500 and -700A600 demonstrated in Figure 6a,b. The short rods might have originated from the accumulation of a strip of crosslinked PIM, as described in Scheme 3. No significant flake-like self-assembled slabs of associated N-GF or CNT were found. With the increasing of the calcination temperature, these crosslinked strips were able to develop into N-GF planes that could have been associated with the thick slabs due to either the polarity provided by iron and nitrogen doping or the formation of covalent bonds between the planes (Figure 6c–e). The formation of Fe, N-doped GF slabs contributed to the 3D GF structure shown in Figure 6e and Figure S2.

Due to the attacking of the NH_3_ molecules, more micro- and mesopores developed on the surfaces of FeNCs after calcination at temperatures above 800 °C.

Most of the Fe-related articles were actually on the surface of the GF slabs, as seen in the enlarged image in Figure 6f, where standing GF slabs are also perceivable and huge pores are present. These pores could accommodate more input O_2_ molecules that were able to make contact with the active centers of Fe-N or various –N-doped carbon regions, catalyzing the ORR at the cathode. Furthermore, the highly conducting GF slabs that behaved as conducting carbon black (CB) in the Pt/C catalyst were capable of introducing more electrons that were transferred from the anode.

### 3.6. TEM

**Figure 7:**
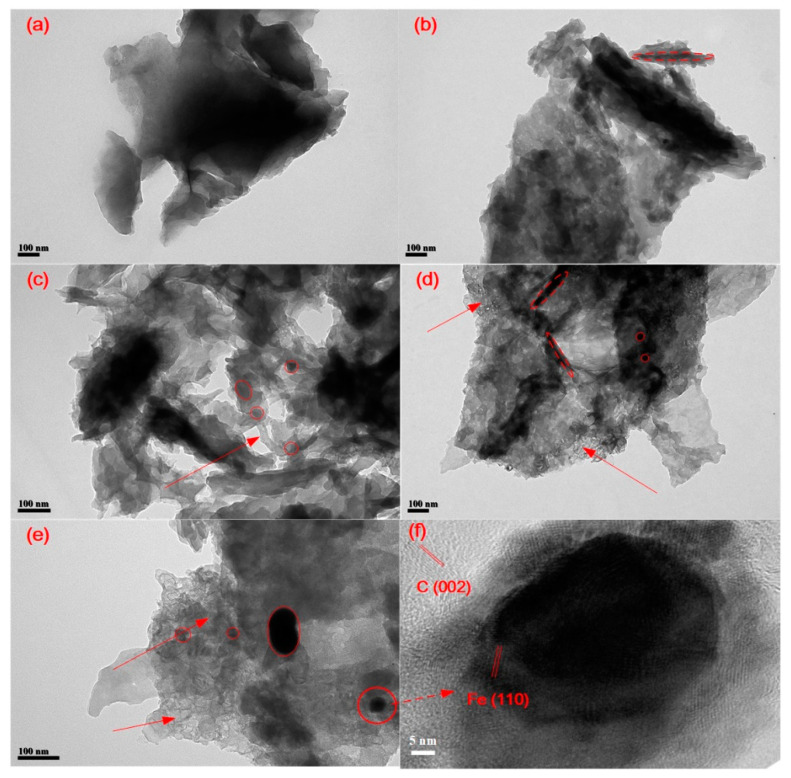
TEM micrographs of (**a**) neat PIM-1000, (**b**) FeNC-700A600, (**c**) FeNC-800A700, (**d**) FeNC-900A800, (**e**) FeNC-1000A900 and (**f**) HR-TEM of the tiny α-Fe particle in FeNC-1000A900.

The TEM micrograph (Figure 7a) of the neat PIM calcined at 1000 °C demonstrates a thick layer morphology with no significant pores or broken sites found in the N-doped carbonaceous matrix. The introduction of iron doping could significantly break the thick layers and generate some short rod-like morphologies at calcination temperatures as low as 700 °C (Figure 7b). The iron doping created larger pores, and the iron atoms acted as the seeds of the formation of CNT from the carbonaceous matrix when the calcination temperature was over 800 °C, as shown in Figure 7c–e. A large number of generated winding carbon nanowires and tiny iron nanoparticles are visible in Figure 7c–e. The iron seed seen in Figure 7f was covered with carbon matrix, demonstrating the presence of the C(002) plane of either GF or CNT. Furthermore, the iron seed is characterized as α-Fe by its (110) plane according to its HR-TEM micrograph in Figure 7f. The full covering of α-Fe by the carbonaceous materials provides further evidence that these covering carbon domains (mainly C(002) planes) were actually growing from the α-Fe seed during calcination (>800 °C).

### 3.7. BET Surface Area

**Figure 8:**
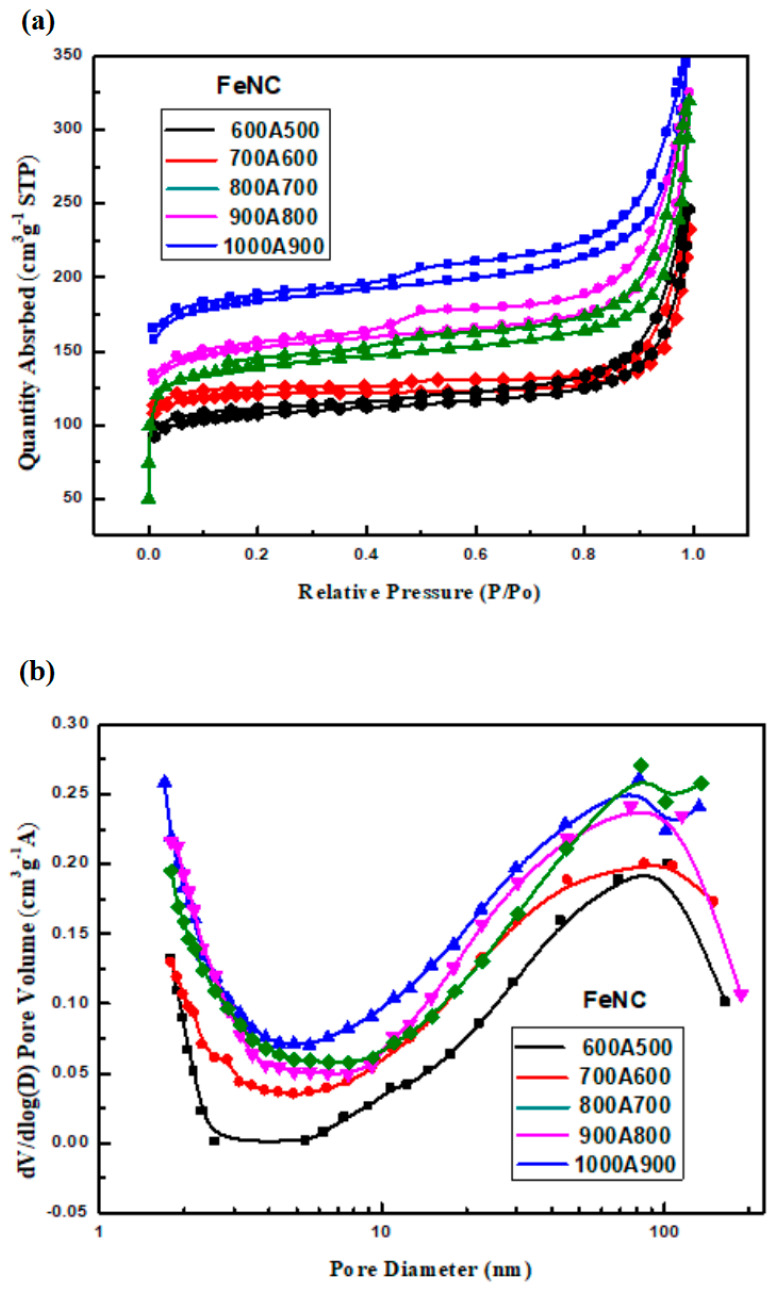
(**a**) N_2_ adsorption–desorption isotherms and (**b**) pore size distribution of FeNCs prepared with different calcination methods.

The type-IV isotherm, which was related to the characteristic N_2_ absorption and desorption curves of the mesopores, can be clearly seen in Figure 8. FeNC-1000A900 (Figure 8a) had a much higher specific volume than the other FeNCs at all relative pressures. Furthermore, in accordance with Figure 8a and Table 3, the surface area (specific volume) became higher and higher with the increasing of the calcination temperature after two-stage calcination in the NH_3_ atmosphere. The collapsing effect caused by NH_3_ at high calcination temperatures could have resulted in increased surface area and the exposure of more Fe-N active sites to O_2_ gas in the cathode. The specific area was increased from 329.0 to 546.6 m^2^·g^−1^ when the FeNC was exposed to increasing calcination temperatures, as shown in Table 3 and Figure 8a.

The pore size distribution, measured via the Barrett–Joyner–Halenda (BJH) method, indicates the presence of both micro- and mesopores, as shown in Figure 8b. The increasing of the surface area with the temperature could have originated from the collapsing power of NH_3_, which not only caused damage on the surfaces but also created more micro- and mesopores. The wide distribution of pore sizes indicates that the FeNCs were able to improve the ORR, since the micropores were able to unveil the active sites and confine the O_2_ inside FeNC catalysts, significantly decreasing the diffusion path. [35]. The average pore sizes created on the FeNC surfaces ranged between 3.7 and 5.0 nm, as listed in Table 3; this allowed more O_2_ molecules to stay inside.

### 3.8. CV and LSV Curve

**Figure 9:**
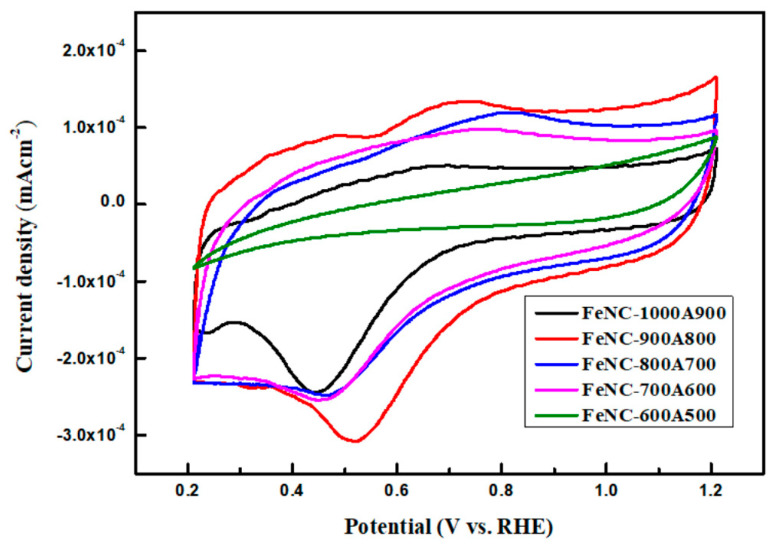
CV curves of FeNCs prepared with different calcination methods.

**Figure 10:**
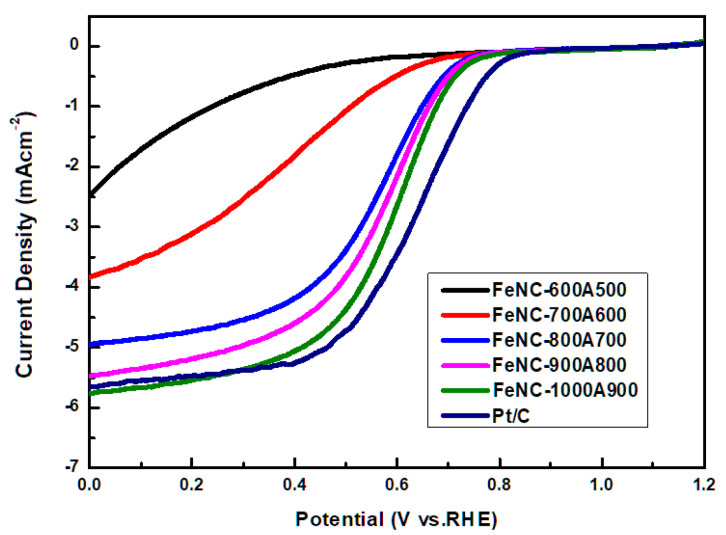
LSV curves obtained from an O_2_-saturated 0.1 M HClO_4_ aqueous solution at a scanning rate of 5 mV/s and a rotation rate of 1600 rpm for FeNCs prepared with different calcination methods.

The electrocatalytic activity of FeNCs, prepared at various temperatures, after acid-leaching was evaluated using the CV and LSV curves of the FeNC catalysts, as shown in Figure 9 and Figure 10.

Except FeNC-600A500, the CV curves of all catalysts demonstrated significant reduction peaks in the O_2_ atmosphere at around 0.4~0.6 V, revealing their abilities, as the cathode catalysts of PEMFC, to cause ORR, as shown in Figure 9.

The LSV curves of all FeNC catalysts could be obtained in an O_2_-saturated 0.1 M HClO_4_ aqueous solution at a scanning rate of 5 mV/s and a rotation rate of 1600 rpm, as illustrated in Figure 10. The reduction current density at 0 voltage ranged from 2.5 to 5.8 mAcm^−2^ when the FeNC was calcined from 500 to 900 after acid-leaching, as can be seen in Figure 10. In particular, the obtained reduction current density (5.8 mAcm^−2^) of FeNC-100A900 was even higher than that of the commercial Pt/C catalyst (5.7 mAcm^−2^), as illustrated in Figure 10. The high reduction current density could be attributed to the presence of more active sites and highly conducting GF (or CNT), as well as the high surface area, which were already discussed in the previous sections.

LSV curves for each FeNC could also be obtained from RDE at different rotating speeds. The potential was selected at the region where the current underwent mixed control by means of both kinetic and mass transfer (diffusion control) and the Koutecký–Levich (K–L) plot was linear, in accordance with Equation (1)

1/I = 1/I_K_ + 1/I_D_ (1)

where:

I_K_—the current contributed by kinetic control

I_D_—the current contributed by diffusion control, which can be expressed in the form of Equation (2):

I_D_ = 0.62 × *A*nFD^2/3^*ν*^−1/6^*C*√ω (2)

A—the geometric area of the disk (cm^2^);

F—Faraday’s constant (C mol ^−1^);

D—the diffusion coefficient of O_2_ in the electrolyte (cm^2^ s^−1^);

*ν*—the kinematic viscosity of the electrolyte (cm^2^ s^−1^);

C—the concentration of O2 in the electrolyte (mol cm^−3^);

ω—the angular frequency of rotation (rad s^−1^);

n—the number of electrons involved in the reduction reaction.

The LSV curves of every type of FeNC are illustrated in Figure S3 and can be used to calculate I_D_. After plotting I^−1^ vs. ω^−1/2^, the K–L lines of FeNC-1000A900 were established, and they are shown in Figure S4a. The slopes of these lines could be used to calculate the numbers of electrons involved in the reduction reaction (n). The electrons transferred for ORR differed from applied voltages and the average value was around 3.85 according to Figure S4b. If the rotating speed became faster than 2500 rpm, both FeNC-1000A900 and -900A800 demonstrated higher reduction currents than that of the commercial Pt/C catalyst, as seen in Figure S3, indicating that it is possible to prepare FeNC at lower temperatures to meet the requirement of gaining a comparable reduction in current density to that of Pt/C. Actually, the ORR phenomenon was already present for FeNC-700A600, as indicated by the CV curve shown in Figure 9. The low reduction current density for FeNCs prepared below 800 °C could be attributed to the morphologies that were significantly related to the performance of the catalysts in the ORR.

The numbers of e-transferred for each catalyst, at different potentials, were calculated, and are listed in Table 4, where it can be seen that the average e-transferred numbers increased significantly with the increasing of the calcination temperature. The average numbers ranged from 3.30 to 3.85, and less than one electron followed the 2-e route (Scheme 2); in other words, the ORR carried out via the 4-e route (Scheme 2) was between 65.0 and 92.5% according to Table 4.

### 3.9. Single Cell Testing

**Figure 11:**
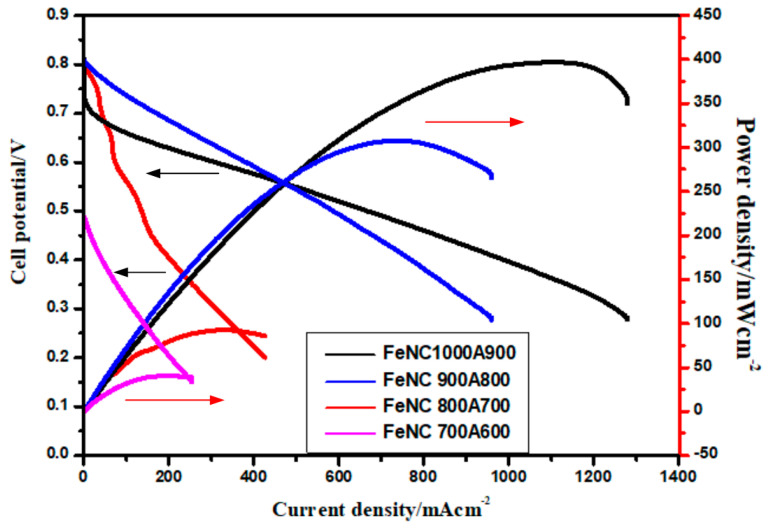
Power and current densities of single cells made of various FeNC cathode catalysts prepared with different calcination methods.

The limited max. power densities (Pmax less than 50 mWcm^−2^) or current densities produced for the single cell with FeNC-700A600 as the cathode catalyst are shown in Figure 11. The Pmax-values grew with the increasing of the calcination temperature (700–1000 °C) from 40 to 400 mWcm^−2^, and increased by 10 times due to the creation of more active centers and the increasing of the surface area. Even when calcined at a lower temperature of 900 °C, the single cell prepared with FeNC-900A800 as the cathode catalyst demonstrated a Pmax value equal to 310 mWcm^−2^. The current density curve even extended to 1000 and 1300 mAcm^−2^ at 0.3 V for FeNC-900A800 and FeNC-1000A900, respectively. The high percentage of electrons (92.5%) that adopted the 4-e route of ORR for FeNC-1000A900 contributed to the higher power and current densities when they behaved as the cathode catalyst, which effectively promoted the ORR without large quantities of hydrogen peroxide being produced.

### 3.10. Durablity Test

**Figure 12:**
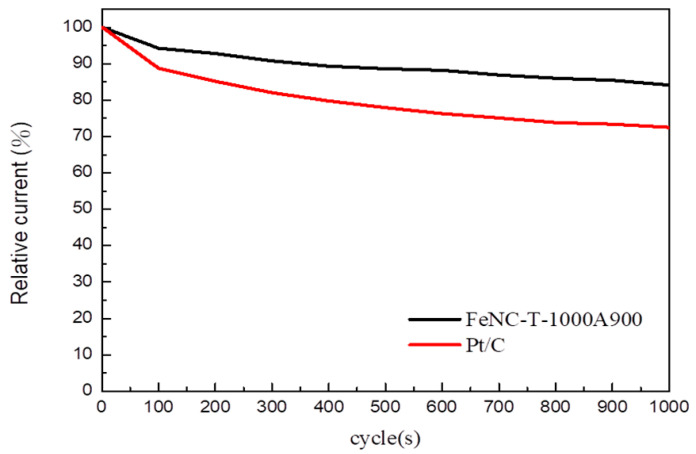
Relative current decay % of the LSV curves vs. cycling numbers recorded in O_2_-saturated 0.1 M HClO_4_ at 1600 rpm and 100 mV/s for the FeNC-1000A900 and Pt/C catalysts, respectively.

A simple test of durability in strong acids was performed by measuring the LSV curves at various cycling times in O_2_-saturated 0.1 M HClO_4_ (aq.), which caused the FeNC catalyst to corrode, resulting in a decrease in the reduction current (Figure 12). The reduction current loss for the FeNC-1000A900 catalyst was only 10% compared to a loss of more than 20% for Pt/C after 1000 cycles, revealing that the non-precious FeNC catalyst was more acid-resistant as compared to Pt/C.

## 4. Conclusions

An aromatic PIM-based Fe- and N-doped organic carbonaceous framework (FeNMOF) was successfully synthesized by high temperature calcination. The non-precious FeNMOF proved to be a promising candidate to replace Pt/C as the most effective cathode catalyst in terms of improving the ORR in the PEMFC.

The complexation between the –Ns of PIM and Fe^2+^ led to the formation of FeNC networks after calcination. The second calcination in the NH_3_ atmosphere, conducted after acid leaching, created high a surface area of 546.6 m^2^·g^−1^ that was composed of high concentrations of both micro- and mesopores, which exposed more Fe-active centers to the O_2_, as characterized by SEM and TEM micrographs for FeNC-1000A900. The high concentration of active centers and the large surface area contributed to a higher reduction current of the cathode and a higher power density of the single cell as compared to the Pt/C catalyst. The non-precious FeNC catalyst even demonstrated higher stability than Pt/C in the durability test performed with 1000 cycles of redox reactions.

In the future, we intend to attempt the preparation of the FeNC catalyst at lower calcination temperatures while avoiding reductions in the current of the cathode and the power density of the prepared single cell.

## Figures and Tables

**Table 1:** Atomic concentration obtained from XPS of FeNCs prepared with different calcination methods.

Catalysts	Atomic Concentration (at%)			
	C	O	N	Fe
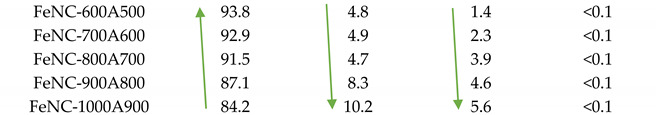				

**Table 2:** Compositions of various –Ns of FeNCs determined by XPS

FeNC Catalysts	N 1s (at%)				
	Pyridinic N	Fe-N	Pyrrolic N	Graphitic N	Pyridine-N Oxide
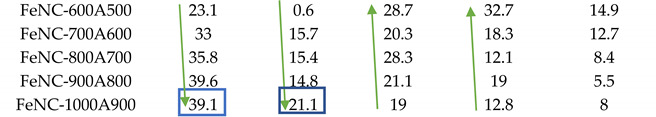					

**Table 3:** Specific area and average pore sizes of FeNCs.

Catalysts	BET Surface Area (m^2^·g^−1^)	Ave. Pore Size (nm)
FeNC-1000A900	564.6	4.95
FeNC-900A800	468.5	4.63
FeNC-800A700	406.6	4.12
FeNC-700A600	364.1	4.29
FeNC-600A500	329.0	3.77

**Table 4:** Numbers of transferred electrons of various FeNCs.

	FeNC	1000A900	900A800	800A700	700A600	600A500
Potential						
0.4018		3.81	3.51	3.70	3.52	3.15
0.35053		3.82	3.93	3.72	3.58	3.24
0.30171		3.84	3.82	3.57	3.64	3.31
0.25044		3.87	3.65	3.54	3.66	3.37
0.20161		3.90	3.61	3.56	3.66	3.42
Ave.		**3.85**	**3.70**	**3.61**	**3.61**	**3.30**
% of 4-e route		**92.5**	**85.0**	**80.5**	**80.5**	**65.0**

## Data Availability

The data presented in this study are available on request from the corresponding author.

## Supplementary Materials

The following are available online at https://www.mdpi.com/article/10.3390/polym13213850/s1, Figure S1: XPS of FeNCs prepared with different calcination methods (a) 600A500 (b) 700A600 (c) 800A700 (d) 900A800 (e) 1000A900, Figure S2: Schematic diagram of the formation of 3D-GF, Figure S3: LSV curves of all FeNC and Pt/C catalysts measured at various rotating speeds, Figure S4: (a) Koutecký-Levich plots of FeNC-1000A900. (b) numbers of electrons transferred during ORR
