# Supplementary material for: Fe, N-Doped Metal Organic Framework Prepared by the Calcination of Iron Chelated Polyimines as the Cathode-Catalyst of Proton Exchange Membrane Fuel Cells
Source: Polymers (Basel). 2021 Nov 8;13(21):3850. doi: 10.3390/polym13213850 (PMC8587573; doi:10.3390/polym13213850)
Supplement: Supplementary file 1 [file polymers-13-03850-s001.zip › polymers-1445524-supplementary.pdf]

Supplementary Materials

Fe, N-doped Metal Organic Framework Prepared by the Calcination of Iron Chelated Polyimines as the Cathode-Catalyst of Proton Exchange Membrane Fuel Cell

Yu-Wei Cheng [1] , Wen-Yao Huang [2], Ko-Shan Ho [3,*], Tar-Hwa Hsieh [3,*], Li-Cheng Jheng [3], Yang-Ming Kuo [3]

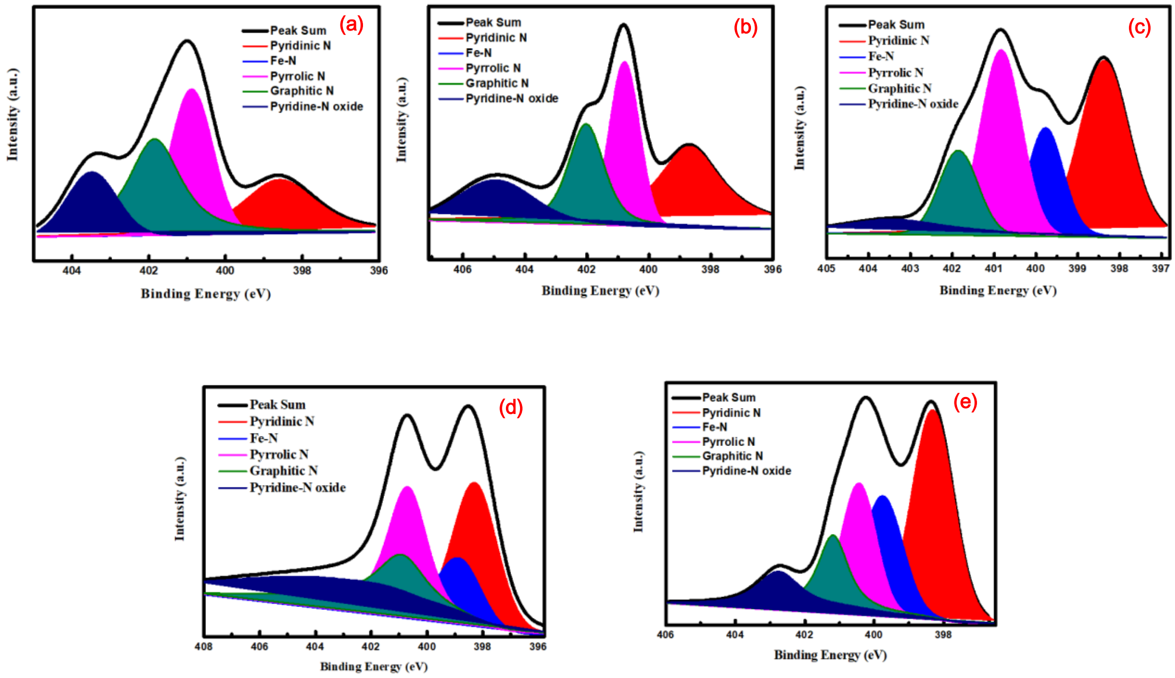

Figure S1- XPS of FeNCs prepared with different calcination methods (**a**) 600A500 (**b**) 700A600 (**c**) 800A700 (**d**) 900A800 (**e**) 1000A900

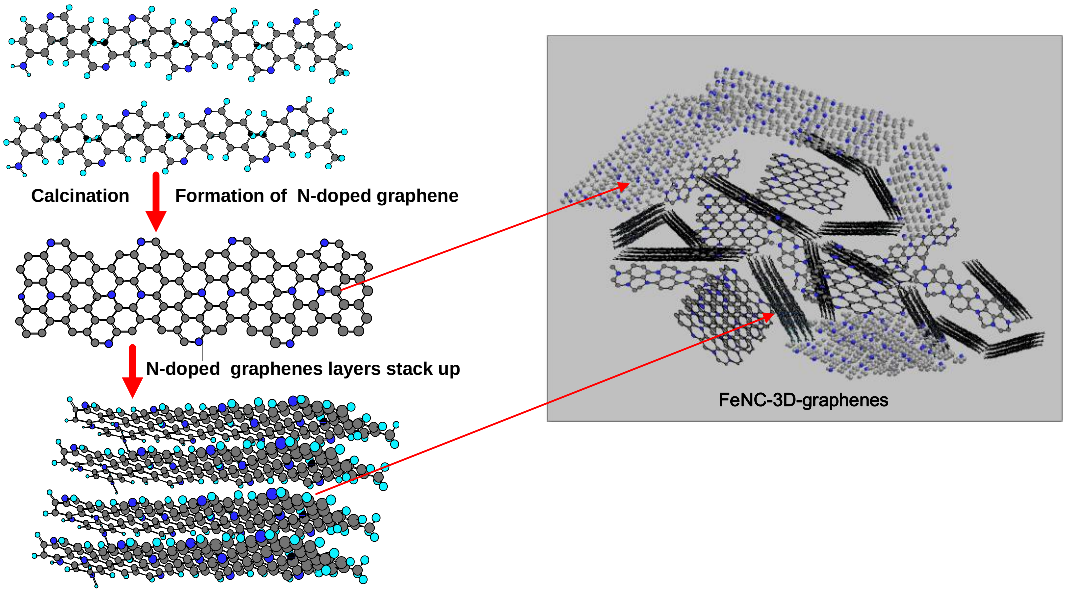

Figure S2- Schematic diagram of the formation of 3D-GF

FeNC

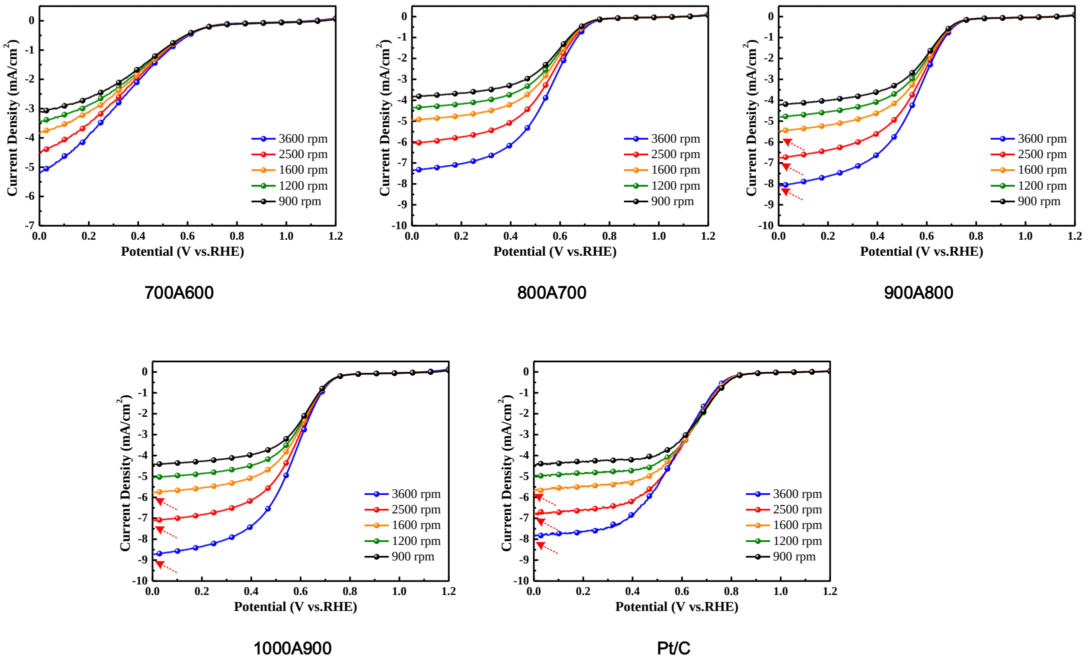

Figure S3-LSV curves of all FeNC and Pt/C catalysts measured at various rotating speeds

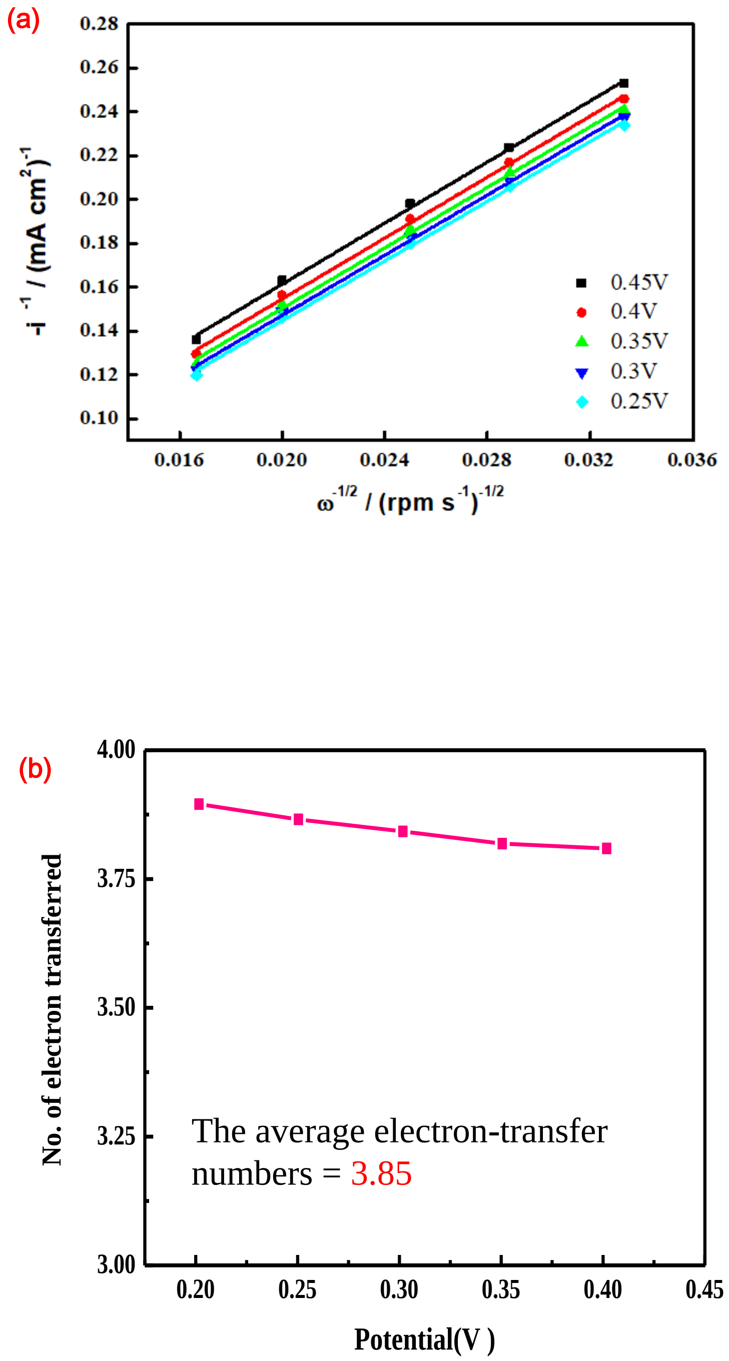

Figure S4- (**a**) Koutecký-Levich plots of FeNC-1000A900. (**b**) numbers of electrons transferred during ORR
